# Ferroptosis-related exosomal non-coding RNAs: promising targets in pathogenesis and treatment of non-malignant diseases

**DOI:** 10.3389/fcell.2024.1344060

**Published:** 2024-02-07

**Authors:** Yiping Zhang, Jun Xie

**Affiliations:** ^1^ School of Life Science, Fudan University, Shanghai, China; ^2^ Wanchuanhui (Shanghai) Medical Technology Co., Ltd., Shanghai, China

**Keywords:** non-coding RNAs, exosomes, ferroptosis, ischemic/reperfusion injury, organ injury

## Abstract

Ferroptosis, an iron-dependent form of programmed cell death, introduces a novel perspective on cellular demise. This study investigates the regulatory network of exosomal non-coding RNAs (ncRNAs), including miRNAs, circRNAs, and lncRNAs, in ferroptosis modulation. The primary goal is to examine the pathological roles of ferroptosis-related exosomal ncRNAs, particularly in ischemic reperfusion injuries. The research reveals intricate molecular interactions governing the regulatory interplay between exosomal ncRNAs and ferroptosis, elucidating their diverse roles in different non-malignant pathological contexts. Attention is given to their impact on diseases, including cardiac, cerebral, liver, and kidney ischemic injuries, as well as lung, wound, and neuronal injuries. Beyond theoretical exploration, the study provides insights into potential therapeutic applications, emphasizing the significance of mesenchymal stem cells (MSCs)-derived exosomes. Findings underscore the pivotal role of MSC-derived exosomal ncRNAs in modulating cellular responses related to ferroptosis regulation, introducing a cutting-edge dimension. This recognition emphasizes the importance of MSC-derived exosomes as crucial mediators with broad therapeutic implications. Insights unveil promising avenues for targeted interventions, capitalizing on the diverse roles of exosomal ncRNAs, providing a comprehensive foundation for future therapeutic strategies.

## 1 Introduction

Ferroptosis is a recently discovered type of programmed cell death (PCD) that is different from apoptosis, necroptosis, or pyroptosis in terms of morphology, biochemistry, and genetics regulation (Li et al., 2023a). This type of PCD is dependent on iron ions for the peroxidation of lipids, which eventually, via the release of toxic lipid peroxides and reactive oxygen species (ROS), results in cell death (Gumilar et al., 2023). Cells undergoing ferroptosis typically manifest necrotic-like morphological alterations, including cell membrane rupture, cytoplasmic swelling, and moderate chromatin condensation (Cui et al., 2021). The primary organelle affected during ferroptosis is the mitochondria, as indicated by mitochondrial shrinkage, increased membrane density, reduced or absent cristae, and rupture of the outer membrane (Ding et al., 2023). On the one hand, induction of ferroptosis might be a promising strategy against cancer cells. At the same time, its inhibition can also be useful in the pathogenesis of some diseases, such as ischemic reperfusion injury, neuronal injury, and autoimmune diseases (Du and Guo, 2022).

Exosomes, minute membrane microvesicles derived from endosomes, have emerged as vital agents in cell-to-cell signaling. Laden with proteins, lipids, and nucleic acids, exosomes play pivotal roles in the development of various diseases. Shed by diverse eukaryotic cells, exosomes influence immune response, signal transduction, and antigen presentation. Their cargo, reflecting the cellular origin and condition, provides predictive insights into conditions like chronic inflammation, cardiovascular ailments, neurodegenerative disorders, lipid metabolic diseases, and tumors (Kalluri and LeBleu, 2020). Despite experimental challenges, exosomes are acknowledged for their potential as clinical indicators, offering valuable information for disease diagnosis and prognosis. Furthermore, the potential of engineered exosomes for precise therapeutic interventions holds promise in treating a spectrum of diseases. In essence, exosomes serve as pivotal messengers in cellular communication, impacting disease processes and presenting avenues for advancements in diagnosis and therapy (Zhang et al., 2019).

Non-coding RNAs (ncRNAs) make up a substantial fraction of cellular RNA and are pivotal in various biological processes. Initially regarded as evolutionary remnants lacking protein-coding capabilities, ncRNAs have garnered attention for their diverse functions in epigenetics, disease, and regulation. Although a portion of RNA is involved in protein translation, a significant majority (80%) is transcribed into ncRNAs. The complex involvement of ncRNAs in gene regulation, development, and maintaining cellular balance has become a central focus in scientific inquiry (Bhatti et al., 2021). MicroRNAs (miRNAs) are short ncRNAs crucial for posttranscriptional gene regulation, implicated in cellular processes and diseases. Circular RNAs (circRNAs), a stable subclass of ncRNAs, influence cellular functions through various mechanisms, such as acting as miRNA sponges. Long non-coding RNAs (lncRNAs), exceeding 200 nucleotides, primarily regulate gene expression and show promise as targets for gene therapies (Bao et al., 2016; Chang et al., 2021; Tabnak et al., 2022; Chen et al., 2023a).

NcRNAs, such as miRNAs, lncRNAs, and circRNAs, play pivotal roles in modulating ferroptosis, a type of cell death associated with iron-driven lipid peroxidation. MiRNAs, short single-stranded RNAs, regulate ferroptosis by targeting essential regulators involved in this process, such as GPX4 and acyl-CoA synthetase long-chain family member 4 (ACSL4). Through binding to specific mRNA sequences, miRNAs influence the expression of these proteins (Wen et al., 2015), thereby contributing to the regulation of ferroptosis (Tabnak et al., 2021; Dai et al., 2022). Conversely, lncRNAs and circRNAs impact ferroptosis by acting as competitive endogenous RNAs (ceRNAs) or sponges for miRNAs (Zhou et al., 2022a; Dabbaghi et al., 2023; Tabnak et al., 2023). These longer non-coding RNAs can sequester miRNAs that would otherwise target and decrease the expression of ferroptosis-related genes. By serving as miRNA decoys, lncRNAs, and circRNAs indirectly control the expression of genes involved in ferroptosis, affecting the cellular susceptibility to this form of cell death. This intricate network of ncRNAs introduces complexity to the regulation of ferroptosis, underscoring their substantial roles in cellular processes and homeostasis (Zheng and Zhang, 2023).

For the first time, this study aims to review the exosomal ncRNAs associated with ferroptosis, unravel the regulatory mechanisms influencing ferroptosis, identify disease-specific signatures, evaluate the impact on cellular functions, and explore the therapeutic potential of targeting these RNAs. We conducted a search in the PubMed database using specific terms and associated keywords, including (Exosome) AND (Ferroptosis) AND (miRNA OR circRNA OR lncRNA). Our inclusion criteria encompassed only experimental studies, while review studies, case reports, editorials, retracted articles, and purely bioinformatic studies were excluded from consideration.

## 2 Overview of exosomal non-coding RNAs

**FIGURE 1:**
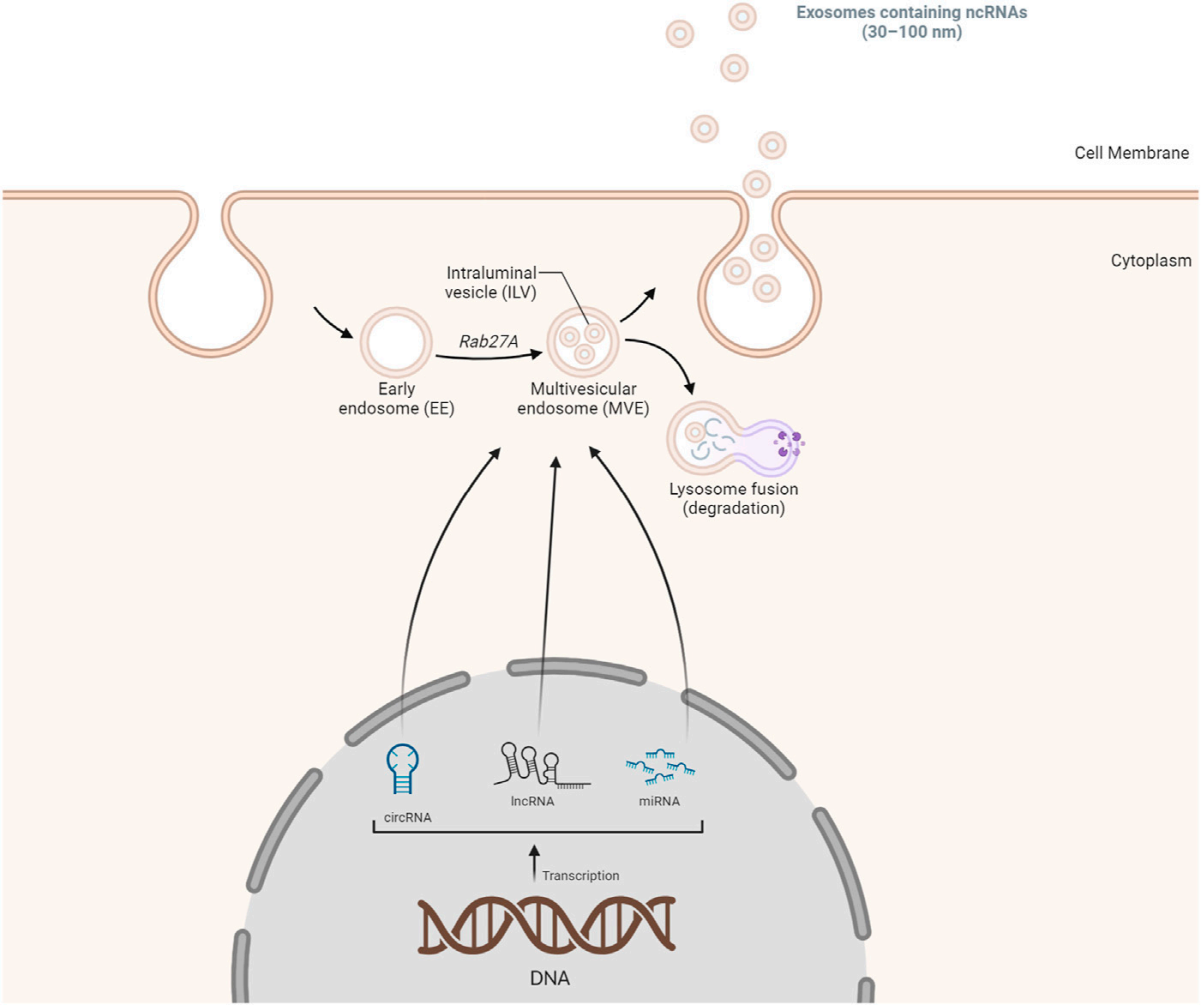
A summary of the biogenesis of exosomal ncRNAs. The biogenesis of exosomes initiates with the formation of double inward cellular membranes, leading to the generation of EEs. Subsequently, non-coding RNA species, including lncRNAs, miRNAs, and circRNAs, are incorporated into these early endosomes. These non-coding RNAs may either be actively transported out from the cellular membrane or subjected to degradation within lysosomes. This intricate process plays a crucial role in the packaging and transportation of various RNA molecules within exosomes, contributing to intercellular communication and regulatory mechanisms.

Exosomes, essential for intercellular communication, are small extracellular vesicles (SEVs, 30–100 nm) carrying diverse biomolecules, including ncRNAs. The formation of exosomes involves a sequence of steps, starting with the double inward folding of cellular membranes. The initial folding generates cup-shaped structures called early-sorting endosomes (ESEs), which can merge with existing ESEs, transforming into late-sorting endosomes (LSEs). A subsequent folding in LSEs leads to multivesicular endosome (MVEs) containing intraluminal vesicles (ILVs), with cytoplasmic components entering ILVs. MVEs can either merge with lysosomes for degradation or release ILVs via exosome shedding through the cell membrane (Figure 1) (Alidadi et al., 2023; Richards et al., 2023). The origin of ncRNAs, including miRNAs, lncRNAs, and circular RNAs, is a pivotal aspect of exosomal composition. MiRNAs, single-strand ncRNAs, are transcribed by RNA polymerase II/III, processed by the microprocessor nuclear complex, and exported to the cytoplasm. Dicer and transactivation response element RNA-binding protein 2 (TARBP2) further process pre-miRNAs, forming miRNA-induced silencing complexes (miRISCs). LncRNAs, primarily produced as messenger RNAs through Pol II/III, undergo post-transcriptional processing and can interact with nucleosomes. Circular RNAs often result from exon and intron circularization during splicing processes (Alidadi et al., 2023). Extracellular vesicles, including exosomes, harbor various RNA types. RNAs can be directed to cytoplasmic endosomes and integrated into MVEs containing ILVs. The resultant structure can fuse with the cell membrane, releasing exosomes containing a diverse array of RNAs (Figure 1). The incorporation of ncRNAs into exosomes can occur through diverse mechanisms, such as passive packaging, specific RNA packaging, motif-mediated packaging, and RNA-RBP mediated packaging. In summary, exosomal ncRNAs, encompassing miRNAs, lncRNAs, and circRNAs, exert multifaceted effects on disease progression (Alidadi et al., 2023). Their involvement spans various pathological conditions, including atherosclerosis, cardiovascular diseases, malignancies, autoimmune diseases, infectious diseases, metabolic diseases, and neurodegenerative diseases. This mechanism underscores the significance of exosomal ncRNAs in cellular communication and disease regulation (Alidadi et al., 2023; Spanos et al., 2023). Interestingly, the delivery of ncRNAs through exosomes has emerged as a novel and promising therapeutic avenue. As natural carriers, exosomes offer a unique platform for the targeted delivery of ncRNAs, such as miRNAs and small interfering RNAs (siRNAs), owing to their inherent ability to protect these fragile molecules from degradation. These ncRNAs play pivotal roles in the regulation of gene expression and cellular processes, and their dysregulation is often implicated in various diseases, including cancer and neurodegenerative disorders. Researchers have successfully loaded ncRNAs into these extracellular vesicles, enhancing their stability and facilitating their delivery to specific cells or tissues by harnessing the natural cargo-carrying capacity of exosomes. Studies have demonstrated the therapeutic potential of exosome-mediated delivery of ncRNAs in modulating key signaling pathways, suppressing oncogenic processes, and mitigating disease progression. This innovative approach holds great promise for the development of targeted and efficient therapeutic interventions, providing a new dimension to the field of RNA-based therapeutics. As advancements continue, exosome-mediated delivery of ncRNAs could revolutionize the treatment landscape by offering precise and tailored solutions for a myriad of diseases (Chen et al., 2021a).

## 3 Overview of ferroptosis

**FIGURE 2:**
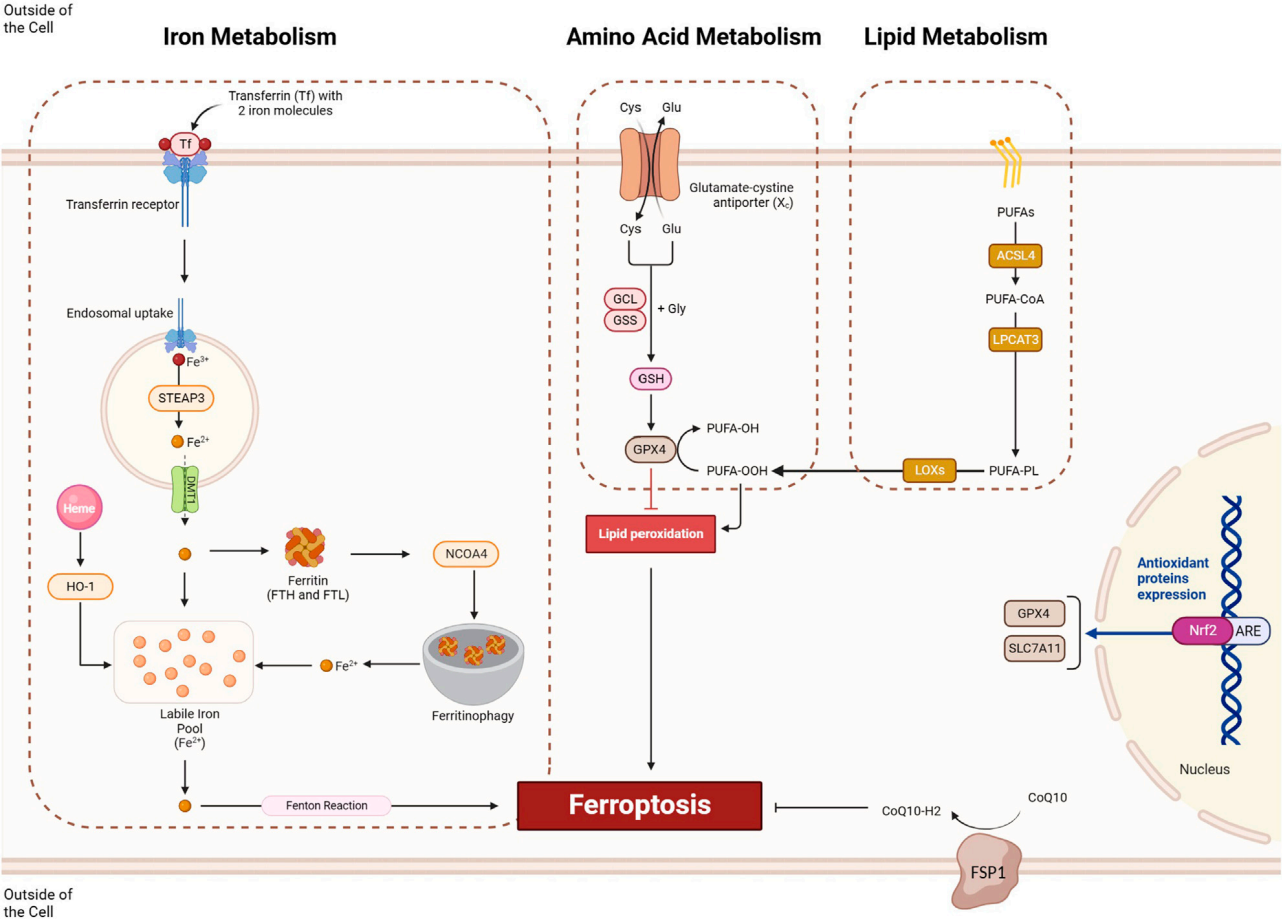
The ferroptosis process involves three key pathways: iron metabolism, amino acid metabolism, and lipid metabolism. Within the iron metabolism pathway, iron ions are taken up through the formation of a complex involving the transferrin receptor and transferrin, incorporating two iron molecules. Subsequently, these iron ions undergo endosomal uptake, where Fe^*3+*^ is converted to Fe^*2+*^ by STEP3 and then transported out by DMT1 from the exosome. Fe^*2+*^ ions can either be stored in ferritin molecules or utilized for ferroptosis. NCOA4-mediated ferritinophagy enhances this process by degrading ferritin molecules, releasing sufficient iron for ferroptosis. In the amino acid metabolism pathway, the proper functioning of System Xc and GPX4 is crucial for preventing ferroptosis in cells. Meanwhile, the lipid metabolism pathways involve the ACSL/LPCAT/LOX axis, which provides lipid peroxides for ferroptosis induction. Conversely, pathways like NRF2 can prevent ferroptosis induction by transcriptionally activating the expression of SLC7A11 and GPX4, contributing to cellular homeostasis.

Ferroptosis is a form of regulated cell death initiated by the disruption of cellular redox balance and marked by the iron-dependent accumulation of lethal lipid peroxides. Three key pathways contribute to ferroptosis (Figure 2): the *amino acid metabolism pathway*, where inhibition of cystine/glutamate exchange transporter System Xc-leads to reduced glutathione and inactivation of GPX4, an enzyme critical for lipid peroxide detoxification; the *lipid metabolism pathway*, involving the peroxidation of polyunsaturated fatty acids (PUFAs), particularly arachidonic acid, and the role of enzymes like ACSL4 and lysophosphatidylcholine acyltransferase (LPCAT3) in promoting lipid peroxidation; and the *iron metabolism pathway*, where redox-active iron catalyzes the formation of damaging free radicals, and iron metabolism genes and ferritin are essential for maintaining cellular iron homeostasis. These interconnected pathways illustrate the mechanisms underlying ferroptosis regulation (Luo et al., 2021a). The modulation of ferroptosis holds promise in various diseases. In cancer treatment, inducing ferroptosis can be beneficial due to its potential to selectively eliminate cancer cells that are sensitive to this form of cell death. Conversely, inhibiting ferroptosis might be advantageous in neurodegenerative diseases, where neuronal cell death is a significant concern (Zhang et al., 2022a), and in conditions involving ischemia-reperfusion injury, where limiting cell death could mitigate damage. Striking the right balance in regulating ferroptosis could offer therapeutic avenues in diverse medical contexts, emphasizing the importance of understanding its mechanisms for targeted interventions.

### 3.1 Amino acid metabolism

#### 3.1.1 System Xc^−^

System Xc^−^ plays a crucial role in ferroptosis by influencing cellular redox balance. System Xc^−^ is a membrane transporter composed of xCT (SLC7A11) and SLC3A2 subunits, responsible for importing extracellular cystine in exchange for intracellular glutamate. Cystine is a precursor for glutathione (GSH) synthesis, a key antioxidant. Inhibition of System Xc^−^ leads to decreased cystine uptake, resulting in GSH depletion (Figure 2). GSH is essential for the activity of GPX4, an enzyme that protects cells from lipid peroxidation. Therefore, System Xc^−^ inhibition indirectly inactivates GPX4, contributing to the accumulation of lethal lipid peroxides and initiation of ferroptosis. The decreased GSH levels disrupt the cellular antioxidant defense, making cells more susceptible to iron-dependent lipid peroxidation and ferroptotic cell death (Liu et al., 2021). The role of ncRNAs in regulating System Xc^−^ is a complex and evolving area of research. Some studies suggest that certain ncRNAs can influence the expression or activity of components within the System Xc-pathway. For instance, miRNAs might bind to the mRNA of System Xc^−^ subunits, modulating their translation and affecting cystine/glutamate exchange. LncRNAs or other types of small RNAs could potentially participate in the regulatory network of System Xc^−^, impacting cellular redox balance and susceptibility to ferroptosis (Xie and Guo, 2021).

#### 3.1.2 GPX4

GPX4, or glutathione peroxidase 4, is a pivotal enzyme responsible for safeguarding cells against oxidative harm. It holds a central role in governing ferroptosis. GPX4 is vital for preventing the destructive peroxidation of membrane lipids by utilizing GSH as a coenzyme to facilitate the reduction of lipid peroxides. In the ferroptosis context, GPX4 can be hindered either indirectly, through the reduction of GSH via System Xc^−^ inhibition, or directly through specific ferroptosis-inducing agents like RSL3. The inhibition of GPX4 leads to the accumulation of lipid peroxides, initiating the ferroptotic process and eventually causing cell death (Seibt et al., 2019). Specifically, GPX4 utilizes GSH as a cofactor to detoxify lipid hydroperoxides, including polyunsaturated fatty acid hydroperoxides (PUFA-OOH), converting them into their corresponding alcohols (PUFA-OH). This enzymatic activity of GPX4 is essential for maintaining the integrity of cell membranes and preventing the accumulation of toxic lipid peroxides. The inhibition or loss of GPX4 activity, as seen in ferroptosis, leads to the accumulation of lipid peroxides and eventual cell death (Figure 2) (Feng and Stockwell, 2018). Targeting GPX4 by miRNAs could be a promising strategy to induce ferroptosis in cancer cells. Since GPX4 is a key regulator that protects cells from lipid peroxidation and oxidative damage, downregulating its expression through miRNA-mediated mechanisms may lead to reduced GPX4 activity and increased susceptibility to ferroptosis. Inducing ferroptosis in cancer cells is a potential therapeutic approach, as it represents a form of regulated cell death that could selectively eliminate cancer cells (Xu et al., 2022).

### 3.2 Iron metabolism

#### 3.2.1 Transferrin

Transferrin (TF) is a glycoprotein located in the cell membrane and a key player in the iron metabolism associated with ferroptosis. It functions as an iron-binding serum protein that is transported into cells through transferrin receptor 1 (TfR1)-mediated endocytosis. Inside the cells, iron-loaded TF contributes to the availability of labile iron, which is fundamental for ferroptosis execution. TF has been identified as an essential component for inducing ferroptotic cell death, and only iron-loaded TF, transported into the cell via TfR1, can exert its corresponding ability to induce ferroptosis. The regulation of TF and its interaction with TfR1 play a crucial role in ensuring a sufficient labile iron pool, contributing to the initiation of ferroptosis (Figure 2) (Lei et al., 2019).

#### 3.2.2 STEAP3

Six-transmembrane epithelial antigen of the prostate 3 (STEAP3) is a metalloreductase, specifically a ferrireductase, located in the endosomal/lysosomal membrane. STEAP3 plays a role in the reduction of ferric (Fe^3+^) iron to ferrous (Fe^2+^) iron, facilitating its release into the cytoplasm (Figure 2) (Chavoshinezhad et al., 2023). This function contributes to the regulation of intracellular iron levels, influencing processes like iron storage and utilization.

#### 3.2.3 DMT1

Divalent metal transporter 1 (DMT1) is responsible for the transport of divalent metals, including iron, across the cell membrane. DMT1 is crucial for the uptake of extracellular iron into the cell, contributing to the maintenance of cellular iron homeostasis. The regulation of DMT1 expression and activity is significant in controlling the influx of iron, a key factor in ferroptosis (Figure 2) (Lee and Roh, 2023).

#### 3.2.4 HO-1

Heme oxygenase-1 (HO-1) is an enzyme involved in the breakdown of heme molecules, converting them into biliverdin, carbon monoxide (CO), and ferrous iron (Fe^2+^) (Figure 2). This process regulates iron and ROS levels in the body. While HO-1 typically acts as a protective mechanism against oxidative stress, it can also induce ferroptosis under certain conditions. The ferroptotic role of HO-1 is linked to iron accumulation, and excessive activation can lead to cytotoxicity due to a rise in labile iron. This results in increased ROS production, causing oxidative damage, lipid peroxidation, and eventual ferroptosis. The dual function of HO-1 depends on the extent of ROS production, determining whether it acts protectively or induces ferroptosis (Chiang et al., 2018). NRF2, also known as nuclear factor erythroid 2-related factor 2, is a key player in controlling the expression of HO-1. In normal circumstances, NRF2 activity is hindered by its interaction with Keap1 (Kelch-like ECH-associated protein 1), leading to NRF2 degradation. However, when the cell encounters oxidative stress, Keap1 undergoes a conformational change, freeing NRF2. The liberated NRF2 moves into the nucleus, where it forms a complex with small Maf proteins and binds to specific regions in the promoter of target genes, including HO-1. This binding initiates the transcription of these genes, facilitating the production of HO-1. HO-1, in response, aids cellular defense by breaking down heme into biliverdin, carbon monoxide (CO), and ferrous iron (Fe2+). The interplay between NRF2 and HO-1 is vital for the cell’s antioxidant response, serving as a critical mechanism to counteract oxidative stress (Song and Long, 2020).

#### 3.2.5 Ferritin

Ferritin, particularly its heavy chain subunit (FTH), assumes a pivotal role in ferroptosis by effectively sequestering and storing iron. In the context of ferroptosis, the equilibrium of iron levels is disrupted, fostering the buildup of labile iron that triggers the production of ROS and subsequent oxidative stress. FTH serves as a protective element against ferroptosis by capturing excess intracellular iron, hindering its involvement in detrimental reactions that contribute to cellular demise. Hence, the control of ferritin expression and functionality stands as a crucial aspect of cellular responses to stimuli inducing ferroptosis. The interplay between ferritin and iron homeostasis significantly influences the susceptibility of cells to ferroptosis (Chen et al., 2020).

#### 3.2.6 NCOA4

NCOA4, known as nuclear receptor coactivator 4, plays a pivotal role in maintaining cellular iron balance through its involvement in ferritinophagy. Ferritinophagy is a selective autophagic process responsible for breaking down ferritin, the cellular protein responsible for storing iron. Functioning as a cargo receptor, NCOA4 facilitates the transportation of ferritin to autophagosomes for subsequent degradation within lysosomes. By promoting ferritinophagy, NCOA4 ensures the controlled release of stored iron, preventing iron overload and the associated oxidative stress. This underscores the critical role of NCOA4 in regulating cellular iron metabolism (Figure 2) (Santana-Codina et al., 2021; Yan et al., 2023).

#### 3.2.7 Fenton reaction

Following the Fenton reaction, labile iron in the iron pool can contribute to generating ROS. The Fenton reaction involves the reaction of hydrogen peroxide (H_2_O_2_) with ferrous iron (Fe^2+^), resulting in the production of hydroxyl radicals (OH) and ferric iron (Fe^3+^). The hydroxyl radicals generated in this process are highly reactive and can cause oxidative damage to various cellular components, including lipids, proteins, and nucleic acids. This oxidative stress, induced by labile iron-mediated ROS production, is implicated in multiple pathological processes and can contribute to cell damage and death, including ferroptosis. In ferroptosis, the dysregulation of iron homeostasis and the accumulation of labile iron play a crucial role in promoting lipid peroxidation and cell death (Ishida, 2018).

### 3.3 Lipid metabolism

In the lipid metabolism pathway, PUFAs go through a series of steps, ultimately leading to the formation of PUFA-OOH. This complex process is guided by enzymes like ACSL4, LPCAT, and lipoxygenases (LOX). It begins with ACSL4 activating PUFAs, turning them into acyl-CoA derivatives (PUFA-CoA) by attaching CoA groups. Next, LPCAT steps in, facilitating the esterification of PUFAs with lysophosphatidylcholine (LPC). This step is crucial for integrating PUFAs into the cellular membrane structure. The process peaks with LOX enzymes, which play a vital role in oxidative modifications. LOX catalyzes the addition of molecular oxygen to PUFA-containing phospholipids (PUFA-PLs), resulting in the creation of PUFA hydroperoxides (PUFA-OOH). These PUFA-OOH molecules become key players in lipid peroxidation, a process linked to ferroptosis. This coordinated dance of biochemical events highlights the profound impact of lipid metabolism on the composition of cellular membranes and the generation of lipid mediators crucial for various cellular processes, including the intriguing phenomenon of ferroptosis (Figure 2) (Yang et al., 2023a).

### 3.4 FSP1 mediaited ferroptosis suppression

Ferroptosis suppressor protein 1 (FSP1), also known as AIFM2, which stands for apoptosis-inducing factor mitochondria-associated 2, emerges as a key player in the realm of ferroptosis. Functioning as a critical component of the coenzyme Q10 (CoQ10) biosynthetic pathway, FSP1 plays a pivotal role in combating lipid peroxidation and oxidative stress. By reducing CoQ10, FSP1 contributes to the overall cellular antioxidant defense, thwarting the progression of ferroptosis. This highlights FSP1 as a potent suppressor of ferroptotic cell death, providing a new avenue for understanding and potentially modulating this regulated cellular demise (Figure 2) (Bersuker et al., 2019).

## 4 Exosomal ferroptosis-related ncRNAs in pathogenesis of human diseases

### 4.1 Ischemic reperfusion injury

Ischemia/reperfusion (I/R) injury occurs when tissues undergo temporary blood supply blockage followed by restoration, leading to damage. miRNAs and lncRNAs play crucial roles in various tissues during I/R injury, regulating stress response and apoptosis. Understanding the roles of these ncRNAs is essential for developing targeted therapies to mitigate I/R injury (Ghafouri-Fard et al., 2020). Exosomal ncRNAs are crucial in regulating ischemia-reperfusion injury through various mechanisms. These ncRNAs can be transferred from donor to recipient cells, influencing key pathways involved in ischemic reperfusion injury. For instance, exosomal miRNAs may suppress the expression of target genes, modulating inflammation, oxidative stress, and apoptosis. LncRNAs carried by exosomes can also act as molecular sponges or decoys, regulating the activity of specific miRNAs (Zhou et al., 2022b). Additionally, exosomal circRNAs may interact with miRNAs and proteins to fine-tune cellular responses. The comprehensive interplay of exosomal ncRNAs in these regulatory processes highlights their potential as therapeutic targets for mitigating ischemic reperfusion injury (Liu et al., 2020). The inhibition of ferroptosis is crucial for reversing I/R injury in various organs. Ferroptosis is implicated in oxidative stress and cell death mechanisms associated with I/R injury in organs such as the heart, brain, kidneys, liver, intestines, lungs, and testicles. The potential therapeutic strategies, such as ferroptosis inhibitors and other compounds targeting this process, indicate that suppressing ferroptosis could be an effective approach to mitigate the severity of I/R injury in these organs (Chen et al., 2021b).

#### 4.1.1 Cardiac ischemic reperfusion injury

I/R injury in the heart occurs when blood flow is temporarily blocked, leading to cellular damage due to insufficient oxygen and nutrients. The subsequent restoration of blood flow paradoxically exacerbates the injury through oxidative stress and inflammation. Exosomal ncRNAs, especially miRNAs, play a regulatory role in this process. These miRNAs, contained in small exosome vesicles, can either protect against or worsen I/R injury by influencing processes like apoptosis, inflammation, and oxidative stress. Understanding the impact of exosomal ncRNAs on I/R injury offers insights into potential therapeutic approaches for managing cardiovascular diseases (Liu et al., 2020; Marinescu et al., 2022).

##### 4.1.1.1 miR-26b-5p

**FIGURE 3:**
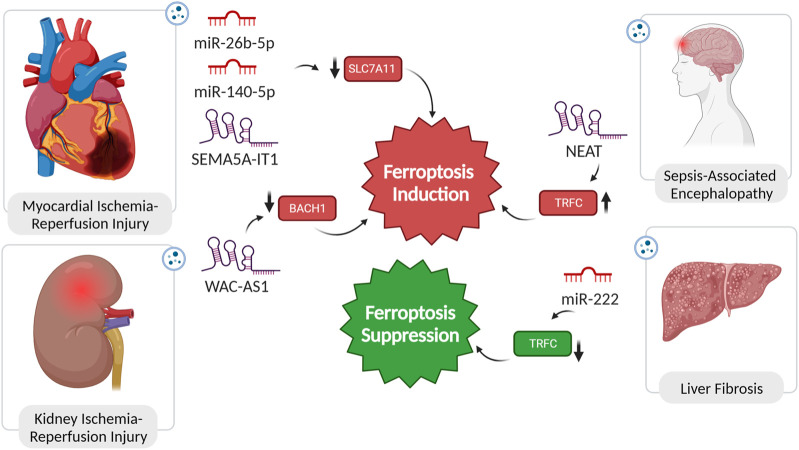
Non-coding RNAs in pathogensis of ischemic reperfusion injuries, encephalopathy, and liver fibrosis. In diseases such as myocardial and kidney injury, as well as sepsis-associated encephalopathy, the dysregulated expression of ncRNAs plays a pivotal role in inducing ferroptosis. This dysregulation alters the expression of ferroptosis regulators, exacerbating the pathological conditions. Conversely, in liver fibrosis, the suppression of ferroptosis is attributed to the functional role of specific ncRNAs. Remarkably, inducing ferroptosis in the context of liver fibrosis has been demonstrated to have a reversing effect on the fibrotic process, highlighting the potential therapeutic significance of modulating ferroptosis through the manipulation of ncRNA expression.

Li et al.’s study reveals a significant depletion in miR-26b-5p expression in exosomes from acute myocardial infarction (AMI) patients (MI-Exo). Increasing miR-26b-5p levels counteract MI-Exo’s suppressive impact on ferroptosis, potentially by modulating SLC7A11 expression, a pivotal regulator of ferroptosis. The observed ferroptotic hallmarks, including reduced ROS, MDA, and Fe^2+^ levels, and increased GSH and GPX4 levels, highlight the comprehensive influence of miR-26b-5p on ferroptosis modulation. Thus, introducing miR-26b-5p mimic into MI-Exo holds promise as a strategic approach for inhibiting ferroptosis, offering a therapeutic avenue for alleviating adversities associated with AMI (Figure 3) (Li et al., 2023b).

##### 4.1.1.2 miR-140-5p

Cardiomyopathy linked to obesity, characterized by cardiac remodeling and dysfunction without concurrent coronary heart disease or hypertension, poses a significant risk and is associated with sudden cardiac death. Upon release of exosomes from adipose tissue macrophages in obese mice, a substantial increase in miR-140-5p levels in cardiomyocytes amplified ferroptosis. The downstream target, SLC7A11, a critical ferroptosis regulator via GSH synthesis modulation, was identified for miR-140-5p. *In vivo* experiments validated that miR-140-5p induces ferroptosis by inhibiting GSH synthesis through interaction with SLC7A11. Suppressing miR-140-5p expression demonstrated alleviation of ferroptosis and improvement in cardiac injury in obesity (Figure 3) (Zhao et al., 2022).

##### 4.1.1.3 SEMA5A-IT1

In post-cardiopulmonary bypass (CPB) surgery, exosomal SEMA5A-IT1 plays a crucial role in protecting against myocardial ischemia/reperfusion (I/R) injury. After CPB surgery, elevated SEMA5A-IT1 levels in circulating sEVs correlate with favorable cardiac outcomes. Transported to cardiomyocytes via sEVs, SEMA5A-IT1 acts as a cytoplasmic miRNA sponge, specifically interacting with miR-143-3p, preventing it from binding to target mRNAs, including BCL2 and SLC7A11. Elevated SEMA5A-IT1 levels protect cardiomyocytes against hypoxia/reoxygenation (H/R) injury, evidenced by increased viability, reduced apoptosis, and inhibition of ferroptosis (Figure 3) (Wu et al., 2022a).

#### 4.1.2 Renal ischemic reperfusion injury

In the context of I/R injury in renal allografts, the exacerbation of damage is facilitated by the crucial involvement of sEVs in delivering the lncRNA WAC-AS1. Enclosed within sEVs, WAC-AS1 contributes to suppressing BACH2, a transcription factor essential for preserving cellular redox balance. BACH2, when localized to the nucleus, serves as an inhibitor of SLC7A11 and GPX4 transcription. Consequently, the reduction in cellular antioxidant defences, orchestrated by sEV-delivered WAC-AS1 and subsequent BACH2 downregulation, sets the stage for ferroptosis induction during renal allograft IRI (Figure 3) (Li et al., 2023c).

### 4.2 Liver fibrosis

Liver fibrosis (LF) is a pathological state marked by the accumulation of collagen and extracellular matrix proteins in response to prolonged liver injury, often resulting in the formation of fibrous scar tissue and impaired liver function. The activation of hepatic stellate cells (HSC) is a key driver of this process. Ferroptosis exhibits a dual role in liver fibrosis. On one hand, excessive hepatic iron and ferroptosis can contribute to the fibrotic process. On the other hand, inducing ferroptosis, specifically in HSCs, has shown promise in mitigating fibrotic scar formation in experimental scenarios. The interplay between ferroptosis and the progression of LF is still not fully understood, and ongoing research aims to unravel the precise mechanisms involved, paving the way for targeted and effective therapies for this liver condition (Pan et al., 2021). In a study, the impact of hepatitis B virus (HBV) infection on HSC activation and LF was investigated. HBV-infected LO2 cells were found to significantly enhance LX-2 cell activation, as evidenced by increased viability, hydroxyproline (Hyp) secretion, and expression of fibrosis markers. Notably, this effect was attributed to exosomes derived from HBV-infected LO2 cells. The administration of GW4869, an exosome generation blocker, abolished the influence on LX-2 cells, confirming the role of exosomes in promoting activation. Moreover, *in-vivo* experiments with mice demonstrated that HBV-infected LO2 cell-derived exosomes promoted LF. Further analysis revealed that miR-222, one of five upregulated miRNAs in HBV-Exo, played a crucial role in enhancing LX-2 cell activation. Overexpression of miR-222 led to increased LX-2 cell activation, while TFRC overexpression counteracted this effect. Importantly, miR-222 was found to inhibit TFRC-induced ferroptosis, suggesting a mechanism by which it promotes LX-2 cell activation (Zhang Q. et al., 2022b).

### 4.3 Sepsis-associated encephalopathy

Sepsis-associated encephalopathy (SAE) is a severe neurological condition linked to sepsis, characterized by altered cognitive function and a range of symptoms from delirium to coma (Sonneville et al., 2023). In a rat sepsis model, sepsis induced blood-brain barrier (BBB) damage and activated ferroptosis in neurons. Exosomes, significantly upregulated, carried the lncRNA NEAT1, suggesting a role in intercellular communication. NEAT1, acting as a ceRNA for miR-9-5p, promoted ferroptosis stress in brain microvascular endothelial cells by facilitating TFRC and GOT1 expression. *In vivo* validation confirmed that increased miR-9-5p, sponged by NEAT1, relieved ferroptosis by suppressing TFRC and GOT1 expression. This unveils a potential mechanism for exacerbating sepsis-associated encephalopathy through lncRNA and miRNA interactions (Wei et al., 2022).

## 5 Exosomal ferroptosis-related ncRNAs as therapeutics

The therapeutic potential of exosomal ncRNAs lies in its ability to deliver circRNA, miRNA, and lncRNA to specific target cells, influencing gene expression and potentially offering novel treatments for diseases. While these studies are still in the early stages and face challenges in terms of bioengineering for effective drug delivery, they hold promise for future clinical applications (Bullock et al., 2015; Li et al., 2021a). Herein, focusing on exosomal ferroptosis-related ncRNAs, we discuss their role as therapeutic methods in different human diseases (Table 1).

**TABLE 1:** Exosomal Ferroptosis-Related ncRNAs for treatment of different diseases.

Type of disease	Name of exosomal ncRNA	Origin	Effect on ferroptosis	Treatment method	Ferroptosis targets	Model	Results	References
MI	miR-26b-5p	Cardiomyocytes	Inhibitor	miR-26b-5p mimic	SLC7A11	*In vivo*	Improved cardiac function	Li et al. (2023b)
MI	lncRNA-Mir9-3hg	BMSCs	Inhibitor	Exosome injection	PRDX6	*In vivo*	Reduced cardiac infarct size, improved cardiac function, and ameliorated ferroptosis	Zhang et al. (2022c)
MI	miR-23a-3p	HUCB-MSC	Inhibitor	Exosome injection	DMT1	*In vivo*	Reduced myocardial injury in AMI mice	Liu et al. (2022)
Stroke	miR-124	M2-type microglia	Inhibitor	M2-exosomes with miR-124-3p overexpression	NCOA4	*In vitro*	Recovered OGD/R-induced HT22 cell injury	Wu et al. (2022b)
Stroke	circBBS2	UC-MSCs	Inhibitor	Exosome injection	miR-494/SLC7A11	*In vivo*	Relieved cerebral I/R injury	Hong et al. (2023)
Stroke	miR-760-3p	ADSCs	Inhibitor	Intranasal exosome injection	CHAC1/GPX4	*In vivo*	Improved the neurobehavior function	Wang et al. (2023)
Stroke	miR-194	BMSCs	Inhibitor	Exosome transfection	Bach/NRF2	*In vitro*	Protected against OGD/R-induced injury	Li et al. (2021b)
Hepatic Ischemia	miR-214-3p	HMSCs	Inhibitor	Exosome injection	COX2	*In vivo*	Attenuated transplanted steatotic liver IRI	Zuo et al. (2023)
Hepatic Ischemia	miR-29a-3p	HMSCs	Inhibitor	Exosome Transfection	IREB2	*In vivo*	Protected against Steatotic Liver IRI	Li et al. (2022a)
Hepatic Ischemia	miR-124-3p	HMSCs	Inhibitor	Exosome injection	STEAP3	*In vivo*	Supressed UVB light-induced vascular injury	Wu et al. (2022b)
Renal Ischemia	lncRNA TUG1	Human urine-derived stem cells	Inhibitor	Exosome injection	ACSL4	*In vivo*	Ameliorated IRI-induced AKI	Sun et al. (2022)
Wound Injury	MiR-17–92	MSCs	Inhibitor	Topical application	GPX4 and SLC7A11	*In vivo*	Improved angiogenesis during wound healing faster wound healing	Nie et al. (2023)
UVB-induced skin injury	circ-Ash1l	ADSCs	Inhibitor	Exosome injection	MiR-700-5p/GPX4	*In vivo*	Enhanced UV-induced skin damage	Zha et al. (2023)
DFU	circ-ITCH	BMSCs	Inhibitor	Subcutaneous exosome injection	NRF2	*In vivo*	Improved wound healing in DFU mice and reduced pathological damage	Shao et al. (2022)
HBV-GN	miR-223-3p	BMSCs	Inhibitor	Exosome transfection	ACSL4	*In vitro*	Protected against ferroptosis induced by HBx overexpression in podocytes	Yang et al. (2023b)
COPD	miR-26a-5p	EPCs of normal patients	Inhibitor	Exosome injection	PTGS2	*In vivo*	Alleviated CS-induced airway remodeling	Liu et al. (2023)
AF	miR-23a-3p	Cardiac fibroblasts	Inducer	Exosome blockade by GW4869	SLC7A11	*In vivo*	Reduced exosome secretion, fibrosis, inflammation, and ferroptosis	Liu et al. (2022)
Liver Fibrosis	miR-222	HBV-infected LO2 cells	Inhibitor	Exosome blockade by GW4869	TFRC	*In vivo*	Reduced ferroptosis	Zhang et al. (2022b)
IBD	mir-129-5p	HMSCs	Inhibitor	Exosome injection	ACSL4	*In vivo*	Improved weight, reduced disease activity index (DAI) scores, restoration of colon length and spleen size	Wei et al. (2023)
IDD	circ_0072464	BMSCs	Inhibitor	Exosome injection	NRF2	*In vivo*	Promoted matrix synthesis and proliferation	Yu et al. (2022)
Sepsis-Induced Lung Injury	miR-125b-5p	ADSCs	Inhibitor	Exosome injection	Nrf2/GPX4	*In vivo*	Reduced pulmonary microvascular endothelial cells ferroptosis	Shen et al. (2023)
Spinal Cord Injury	lncGm36569	MSCs	Inhibitor	Exosome injection	miR-5627-5p/FSP1	*In vivo*	Improved behavioral scores, reduced inflammation	Shao et al. (2022)

### 5.1 Myocardial ischemic reperfusion injury

The long non-coding RNA Mir9-3hg, abundant in bone marrow mesenchymal stem cells (BMSCs)-derived exosomes, emerges as a key player in mitigating I/R injury-induced ferroptosis in cardiomyocytes. In response to H/R, Mir9-3hg expression decreases in cardiomyocytes, while BMSCs-derived exosomes enriched with Mir9-3hg significantly counteract this reduction. Functionally, Mir9-3hg promotes cell proliferation and diminishes iron ion concentration, ROS levels, and ferroptosis markers in H/R-treated cardiomyocytes. Mechanistically, Mir9-3hg acts as a negative regulator of Pum2, an RNA-binding protein, which, in turn, modulates the expression of peroxiredoxin 6 (PRDX6), a known suppressor of ferroptosis. BMSCs-Exo treatment in an I/R mouse model substantiates these findings, revealing a reduction in cardiac infarct size, improved cardiac function, and ameliorated ferroptosis (Zhang et al., 2022c). Exosomes from human umbilical cord blood mesenchymal stem cells (HUCB-MSCs) carrying miR-23a-3p demonstrate promising therapeutic prospects for AMI in mice by counteracting ferroptosis. AMI triggers an elevation in DMT1 expression, instigating ferroptosis in myocardial tissue. *In vitro* analyses show that administering these exosomes to myocardial cells undergoing H/R significantly impedes ferroptosis, enhancing cell viability, diminished apoptosis, and regulation of oxidative stress indicators (Song et al., 2021). Lastly, exosomal miR-210-3p, notably enriched in hypoxia-conditioned exosomes (H-exo), has been implicated in the inhibition of ferroptosis in H/R (hypoxia/reoxygenation)-induced H9C2 myocardial cells. It has been revealed these exosomes facilitates the direct transfer of this microRNA to H9C2 cells under H/R conditions. TFRC as a specific target of miR-210-3p, was identified, which establishes a molecular link between the microRNAs and the regulation of ferroptosis. The delivery leads to the downregulation of TFR expression in H/R-treated H9C2 cells, effectively inhibiting ferroptosis (Lei et al., 2022).

### 5.2 Ischemic stroke

Exosomal ncRNAs have emerged as influential mediators in the pathogenesis of ischemic stroke, a leading global cause of mortality. The dynamic microenvironmental changes within the central nervous system (CNS) contribute to the complexity of CNS diseases, and exosomes, released from neurons or glial cells, play a crucial role in intercellular communication. These exosomal ncRNAs have been implicated in stroke pathogenesis. For instance, some exosomal ncRNAs have the potential to induce or prevent brain ischemic injury. Elevated levels of specific exosomal ncRNAs (e.g., miR-134) in stroke patients correlate positively with inflammatory markers, suggesting their involvement in intercellular brain injury. Conversely, certain exosomal ncRNAs (e.g., miR-124) exhibit neuroprotective potential by influencing processes such as angiogenesis (Wang et al., 2022). Ferroptosis plays a significant role in the development and progression of strokes. In the aftermath of a stroke, whether caused by insufficient blood supply or bleeding in the brain, increased BBB permeability allows iron-containing substances to enter the brain. Thus, inhibiting ferroptosis seems promising as a way to reverse ischemic stroke-induced cell death. A team of researchers explored the protective function of exosomes derived from M_2_-type microglia (M_2_-exosomes) in countering cytotoxicity induced by oxygen-glucose deprivation/reoxygenation (OGD/R) in mouse HT22 cells, a model representing I/R brain injury. M_2_-exosomes played a crucial role in mitigating OGD/R-induced damage in HT22 cells by delivering miR-124-3p and targeting NCOA4, leading to ferritinophagy suppression (Xie et al., 2023). The therapeutic role of umbilical cord mesenchymal stem cells (UC-MSCs) derived exosomal circBBS2 in countering ferroptosis and relieving cerebral I/R injury is elucidated through several mechanisms. In H/R model mimicking ischemic stroke, exosomal circBBS2 protects against ferroptosis by sponging miR-494, a miRNA downregulated in H/R-challenged cells. In addition, SLC7A11 was identified as a target gene regulated by miR-494, implicating the miR-494/SLC7A11 axis in ferroptosis modulation. In a rat model of cerebral I/R injury, these exosomes have shown therapeutic potential by reducing infarct size, alleviating neurological deficits, and mitigating pathological damage. This suggests that the protective effects are mediated through the regulation of ferroptosis-related pathways, offering a promising avenue for the treatment of cerebral I/R injury (Hong et al., 2023).

**FIGURE 4:**
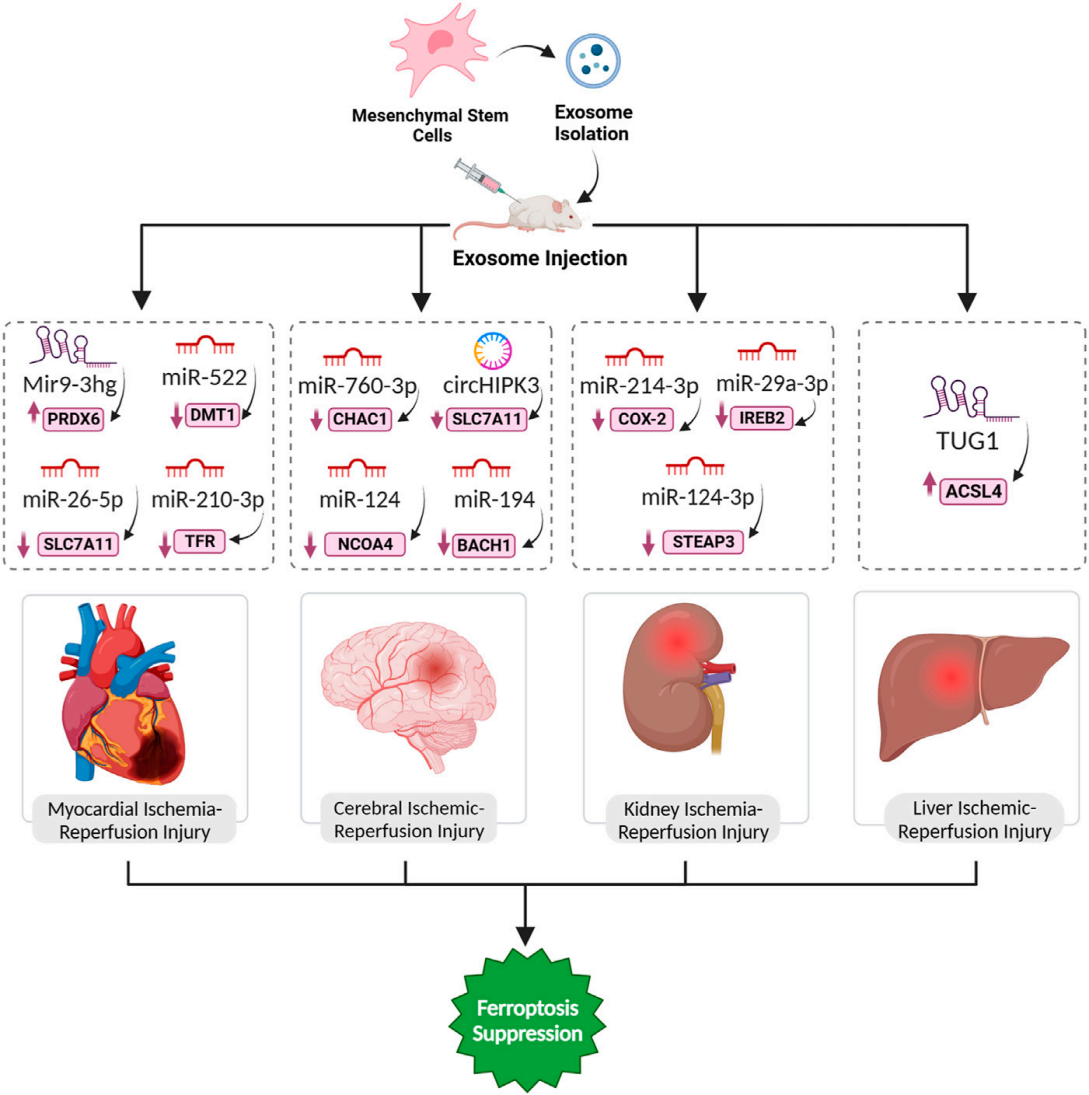
Exosomal ferroptosis-related ncRNAs for treatment of organ ischemic reperfusion injury. The injection of exosomes carrying the aforementioned ncRNAs holds promise as a therapeutic intervention to prevent cell death mediated by ferroptosis. These exosomes, enriched with specific ncRNAs, can effectively suppress the expression of ferroptosis inducer genes. By doing so, they contribute to the inhibition of ferroptosis, ultimately facilitating a reversal of the pathological conditions.

Anti-CHAC1 refers to a therapeutic strategy targeting CHAC1, a key gene associated with ferroptosis in ischemic stroke. A study focused on designing and producing exosomes with anti-ferroptosis properties derived from adipose-derived mesenchymal stem cells (ADSC-Exo) for treating ischemic brain injury. Bioinformatic analysis identified CHAC1 as a crucial gene in the ferroptosis process in ischemic stroke. miR-760-3p, which can inhibit CHAC1 expression, was predicted to be abundant in ADSC-Exo. ADSC-Exo were successfully isolated and shown to be efficiently delivered to the brain through intranasal administration. In a mouse model of I/R, intranasal administration of ADSC-Exo improved neurobehavioral function and mitigated ferroptosis-related outcomes, including the downregulation of CHAC1 expression. Additionally, ADSC-Exo treatment increased the expression of GPX4, an anti-ferroptotic protein, and reduced the expression of ACSL4, a pro-ferroptotic protein. Further experiments demonstrated that miR-760-3p in ADSC-Exo played a role in inhibiting ferroptosis not only by targeting CHAC1 (Figure 4) but also by modulating the levels of GPX4 and ACSL4 in neurons (Wang et al., 2023).

Similarly, in human brain microvascular endothelial cells (HBMECs), OGD/R downregulates miR-194, and its overexpression alleviates cell injury, targeting and downregulating Bach1. The Nrf2/HO-1 pathway, implicated in oxidative stress, is modulated by miR-194, enhancing cell viability. MSC-derived exosomes with miR-194 further enhance protective effects, promoting cell viability, migration, and inhibiting ferroptosis (Li et al., 2021b).

### 5.3 Hepatic ischemic reperfusion injury

Ferroptosis plays a vital role in the pathogenesis of liver injury during IRI. The regulatory impact of iron on inflammation and oxidative damage in hepatic IRI is highlighted, suggesting that iron overload is a critical factor triggering ferroptosis in this context. Thus, the exploration of ferroptosis in hepatic IRI opens avenues for potential therapeutic strategies targeting this specific form of cell death (Luo et al., 2021b). sEVs derived from HO-1 modified BMSCs (HM-sEVs) emerge as a promising therapeutic target for alleviating I/R injury in transplanted steatotic livers. The protective impact of HM-sEVs is attributed to their ability to suppress ferroptosis through the delivery of miR-214-3p, which directly targets and downregulates cyclooxygenase 2 (COX2), a key regulator of ferroptosis. COX2, in turn, modulates the cysteine/GSH/GPX4 axis, which is crucial for cellular antioxidant defense. In both *in vitro* and *in vivo* experiments, HM-sEVs effectively reduce ferroptosis markers, including 4-HNE, ROS, MDA, and Fe^2+^, while enhancing the expression of the ferroptosis suppressor GPX4. The *in vivo* application of HM-sEVs in liver transplantation using steatotic donor livers demonstrates significant attenuation of IRI, reduced tissue injury, and improved mitochondrial morphology. Notably, the knockdown of miR-214-3p in HM-sEVs compromises their protective effects, underscoring the crucial role of the miR-214-3p–COX2 axis in mediating the therapeutic potential of HM-sEVs in preventing ferroptosis-related damage in transplanted steatotic livers (Zuo et al., 2023). In the landscape of steatotic liver I/R injury, exosomal-derived miR-29a-3p emerges as a pivotal player, exerting regulatory control over the ferroptotic process. This miRNA specifically targets iron response element-binding protein 2 (IREB2), a linchpin in cellular iron homeostasis. Ptgs2, integral to lipid peroxidation, experiences a reduction in expression, curbing the initiation of ferroptotic cascades. Simultaneously, the levels of ferroptosis suppressors, such as GPX4, witness an augmentation, reinforcing cellular antioxidant defenses (Li et al., 2022a). A study demonstrated that exosomes from BMMSCs modified with HO-1 inhibit ferroptosis and reduce IRI in steatotic grafts by delivering miR-124-3p. When transplanted into steatotic livers, these exosomes enter hepatocytes, leading to the downregulation of Steap3 (Figure 4). This targeted inhibition of Steap3 results in a reduction of lipid peroxidation, Fe^2+^ accumulation, and overall ferroptotic cell death (Wu et al., 2022b).

### 5.4 Renal ischemic reperfusion injury

Researchers have demonstrated that lncRNA TUG1, when carried in exosomes derived from human urine-derived stem cells (USCs), plays a crucial role in inhibiting ferroptosis in the context of I/R-induced acute kidney injury (IRI-AKI). The USC-Exo effectively ameliorate IRI-AKI, as evidenced by reduced serum markers, improved kidney morphology, and altered levels of ferroptosis-related factors through suppressing the expression of ACSL4 (Figure 4), a key contributor to ferroptosis, through a mechanism involving the interaction between TUG1 and RNA-binding protein SRSF1. This interaction, in turn, enhances the stability of ACSL4 mRNA, ultimately inhibiting ferroptosis in renal cells (Sun et al., 2022).

### 5.5 Wound injury

**FIGURE 5:**
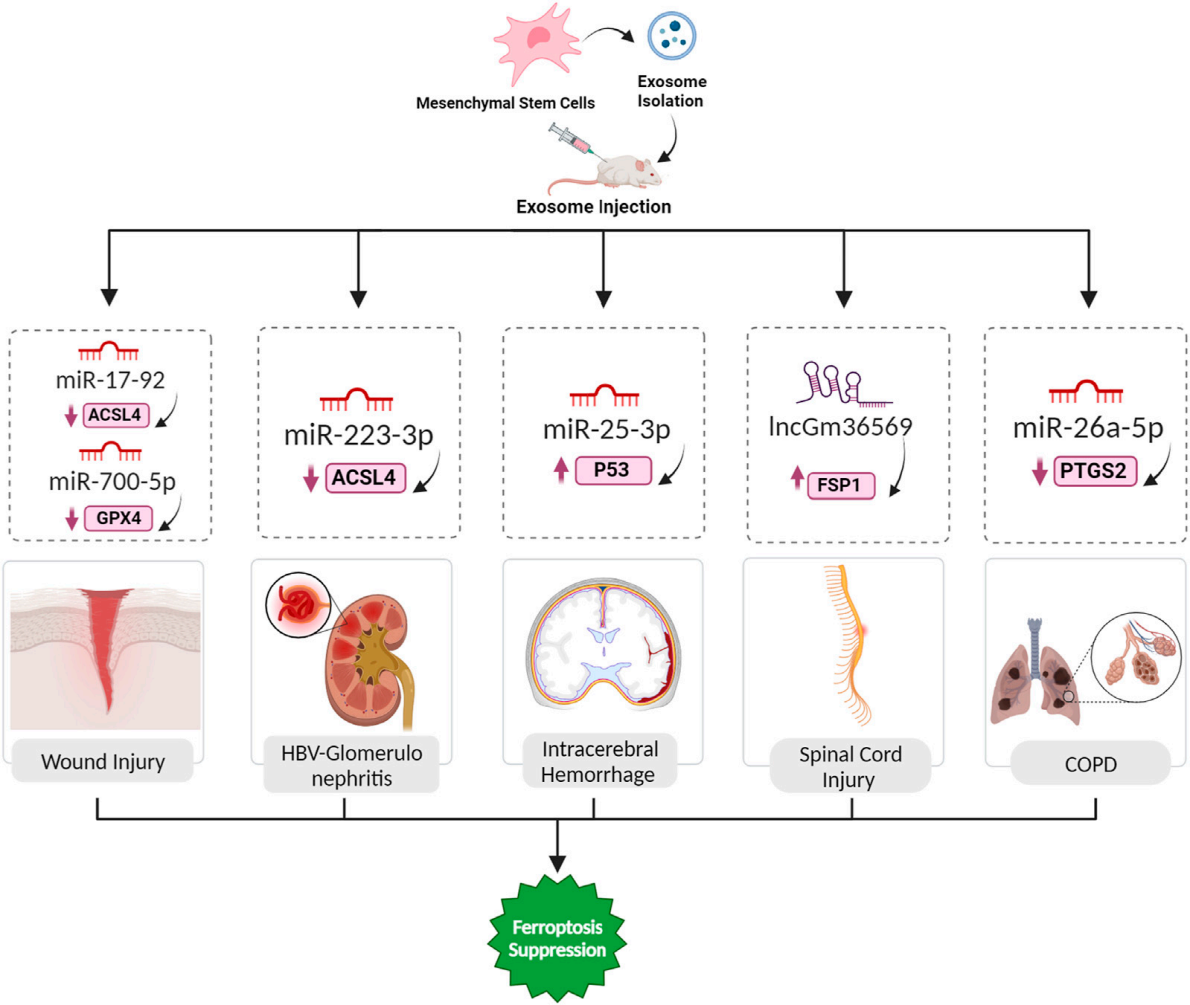
Exosomal ferroptosis-related ncRNAs for treatment wound injury, HBV-GN, ICH, SCI, and COPD. The injection of exosomes carrying the aforementioned ncRNAs holds promise as a therapeutic intervention to prevent cell death mediated by ferroptosis. These exosomes, enriched with specific ncRNAs, can effectively suppress the expression of ferroptosis inducer genes. By doing so, they contribute to the inhibition of ferroptosis, ultimately facilitating a reversal of the pathological conditions.

Ferroptosis emerges as a significant player in wound healing, as indicated by its involvement in various types of wounds. In diabetic wounds, abnormal iron metabolism and increased oxidative stress contribute to ferroptosis, and ferroptosis inhibitors show promise in promoting healing (Li et al., 2023d). Similarly, excessive irradiation triggers ferroptosis in fibroblasts, impeding wound recovery. UV-driven wounds resulting from prolonged UV exposure exhibit ferroptosis-induced cell death in keratinocytes, which can be mitigated by ferroptosis inhibitors. These findings underscore the interplay between ferroptosis and wound healing processes, suggesting that interventions targeting ferroptotic pathways hold therapeutic potential for enhancing recovery in diverse wound types (Bi et al., 2023). MSC-Exos, characterized by a phospholipid bilayer structure and low immunogenicity, contain miR-17-92 cluster, demonstrating its importance in the regenerative capacity of MSCs. Application of these exosomes accelerates cutaneous wound healing in mouse models, fostering faster closure and enhanced angiogenesis. MiR-17-92 protects wounds against ferroptosis by downregulating ACSL4 (a contributor to ferroptosis) and upregulating GPX4 and SLC7A11 (suppressors of ferroptosis). At the cellular level, these exosomes shields endothelial cells from erastin-induced ferroptosis, and within them, miR-17-92 enhance endothelial cell proliferation, migration, and invasion (Nie et al., 2023). Likewise, the regenerative effects of exosomes derived from ADSCs in repairing skin damage induced by ultraviolet B (UVB) light exposure have been investigated. UV overexposure is a significant contributor to skin diseases, including sunburn, mediated by UV-induced oxidative stress, and persistent lesions can lead to various skin disorders, including cancer. Ferroptosis, is implicated in UV-induced skin injuries. It has been demonstrated that exosomes from ADSCs inhibit UVB light-induced vascular injury by reversing ROS and inflammatory factor expression. Additionally, a specific circular RNA, circ-Ash1l, delivered by these exosomes as a key mediator of their therapeutic effects was identified. The overexpression of circ-Ash1l is associated with improved skin damage, and its downstream targets, miR-700-5p and GPX4, play crucial roles in mitigating UV-induced endothelial progenitor cell (EPC) damage (Figure 5) (Zha et al., 2023). Lastly, the potential therapeutic effects of bone marrow stromal cell-derived exosomes enriched with circ-ITCH in the context of diabetic foot ulcer (DFU) wound healing were also investigated. Researchers employed a DFU mice model and hyperglycemic human umbilical vein endothelial cells (HUVECs) to explore the involvement of ferroptosis in DFU progression. Overexpression of circ-ITCH was found to alleviate ferroptosis and promote angiogenesis in HG-treated HUVECs. Furthermore, exosomes derived from BMSCs were investigated for their impact on ferroptosis and angiogenesis in HG-treated HUVECs. These exosomes, particularly those overexpressing circ-ITCH, were found to inhibit ferroptosis and enhance angiogenesis. In more details, circ-ITCH interacts with the TAF15 protein, positively regulating its expression. Additionally, TAF15 was found to alleviate ferroptosis and promote angiogenesis in HUVECs by activating the Nrf2 signaling pathway. The therapeutic potential of exosomal circ-ITCH was further demonstrated *in vivo*, where it promoted wound healing in DFU mice, reduced pathological damage, and regulated ferroptosis markers (Chen et al., 2023b).

### 5.6 Glomerulonephritis

Hepatitis B virus-associated glomerulonephritis (HBV-GN) arises as a complication of chronic hepatitis B infection, wherein immune complexes generated by the HBV accumulate in the kidney glomeruli, causing diverse forms of glomerulonephritis (Yoo et al., 2015). The protective mechanism of MSC-derived exosomes and their miRNAs in the context of HBV-GN involves the regulation of ferroptosis. MSCs release extracellular vesicles, including exosomes, which encapsulate miRNAs such as miR-223-3p. In HBV-GN, the pathogenic influence of HBV is known to induce ferroptosis in kidney cells, contributing to renal damage. A demonstrated that exosomes from BMSCs can be internalized by renal podocytes. Upon exposure to HBV-induced stress, these BMSC-derived exosomes, containing miR-223-3p, effectively attenuate ferroptosis in podocytes. The delivered miR-223-3p plays a vital role in this protective mechanism, potentially by modulating the expression of key ferroptosis-related molecules, including upregulating GPX4 and SLC7A11 while downregulating ACSL4 (Figure 5) (Yang et al., 2023b).

### 5.7 Intracerebral hemorrhage

Ferroptosis is implicated in various acute CNS injuries, including intracerebral hemorrhage (ICH). In ICH, iron overload contributes to secondary brain injury, and GPX4 inactivation is observed. Ferroptosis inhibitors, including Fer-1, DFO, Trolox, and NAC, show promise in alleviating cell death in experimental ICH models. The role of ferroptosis in these CNS injuries highlights its potential as a therapeutic target, emphasizing the need for further research to elucidate the complex interplay between ferroptosis and other cell death pathways in diverse neurological contexts (Shen et al., 2020). Exosomes derived from the plasma of young healthy individuals were found to protect against ferroptosis after ICH. Exosomes were successfully extracted and identified, exhibiting typical features, and their distribution and internalization in the hemorrhagic brain were assessed. Behavioral tests demonstrated that intraventricular injection of these exosomes significantly promoted locomotion recovery in ICH mice. Histological analyses revealed that the exosomes reduced brain injury and cell ferroptosis after ICH. Further experiments demonstrated that miR-25-3p treatment mirrored the effects of exosomes on behavioral recovery, and both exosomes and miR-25-3p suppressed the reduction of GPX4 intensity. Mechanistically, miR-25-3p was shown to counteract ferroptotic injury by regulating the P53/SLC7A11/GPX4 signaling pathway after ICH (Figure 5) (Yang et al., 2023c).

### 5.8 Spinal cord injury

Spinal cord injury (SCI) results in motor and sensory impairments, with traumatic SCI often caused by external forces. In secondary SCI, ferroptosis, characterized by severe lipid peroxidation and disrupted iron metabolism, plays a significant role. Studies using ferroptosis inhibitors and interventions targeting related pathways show promise in reducing ferroptosis, promoting motor recovery, and enhancing neurological function after SCI (Li et al., 2022b). Exosomes derived from MSCs, containing ncRNAs like lncGm36569, offer a promising treatment avenue for acute spinal cord injury (ASCI) by targeting ferroptosis. These exosomes play a pivotal role in alleviating ferroptosis in neuronal cells. In ASCI, secondary injuries worsen neural damage, causing motor and sensory dysfunction. Through the lncGm36569/miR-5627-5p/FSP1 axis, MSC-derived exosomes exhibit a neuroprotective effect by suppressing ferroptosis (Figure 5). In ASCI mice treated with these exosomes, significant enhancements in locomotor function, reduced oxidative stress, and restrained inflammation were observed. This approach introduces a novel therapeutic strategy for ASCI, highlighting the regulatory impact of exosomal ncRNAs on promoting neuronal cell survival and functional recovery (Shao et al., 2022).

### 5.9 COPD

Chronic obstructive pulmonary disease (COPD) is a severe respiratory condition associated with disturbed iron equilibrium. Recent investigations indicate that ferroptosis has a key role in the progression of COPD. Various factors, including reactive oxygen species, cigarette smoke, and particulate matter, can induce ferroptosis in COPD. Targeting pathways linked to ferroptosis, such GPX4, hold the potential for alleviating COPD-related damage. The interconnection between ferroptosis and COPD introduces a novel perspective for precise therapeutic strategies (Meng et al., 2023). A study aimed to investigate the impact of exosomes on COPD by influencing ferroptosis in bronchial epithelial cells (BECs) and the associated mechanisms. The researchers extracted EPCs and EPC-Exo from peripheral blood samples of normal subjects and COPD patients. They established a COPD model in animals and treated human BECs with cigarette smoke extract (CSE) to create a COPD cell model. It was found that EPCs alleviated CSE-induced ferroptosis in BECs by transporting Exo *in vitro* and demonstrated the same effect in a mouse model. CSE-induced ferroptosis promoted the epithelial-mesenchymal transition (EMT) of BECs. The PTGS2/PGE2 pathway was implicated in CSE-induced ferroptosis in BECs, and miR-26a-5p targeting PTGS2 inhibited ferroptosis (Figure 5) (Liu et al., 2023).

### 5.10 Atrial fibrillation

**FIGURE 6:**
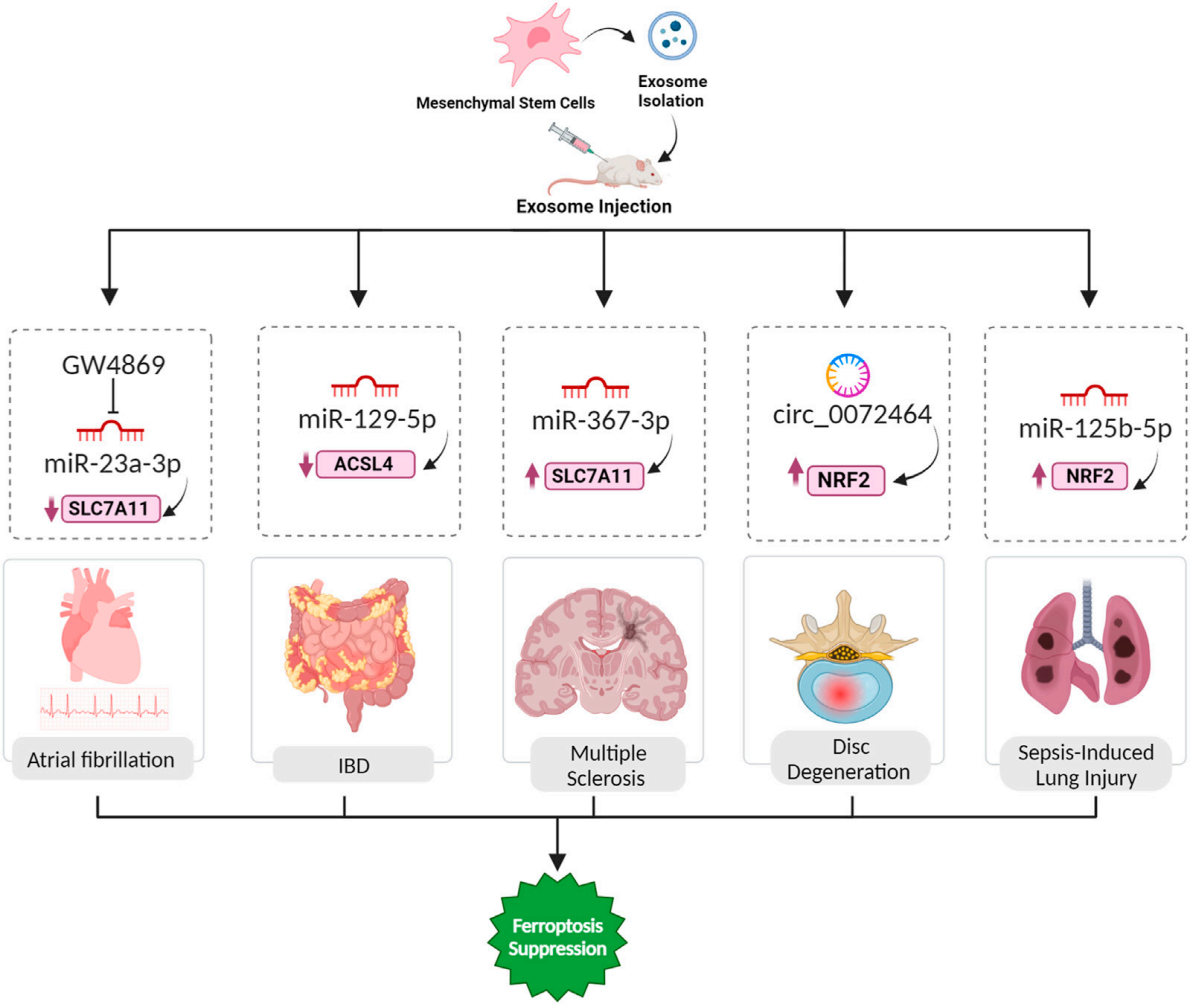
Exosomal ferroptosis-related ncRNAs for treatment of AF, IBD, MS, IDD, and sepsis-induced lung injury. The injection of exosomes carrying the aforementioned ncRNAs holds promise as a therapeutic intervention to prevent cell death mediated by ferroptosis. These exosomes, enriched with specific ncRNAs, can effectively suppress the expression of ferroptosis inducer genes. By doing so, they contribute to the inhibition of ferroptosis, ultimately facilitating a reversal of the pathological conditions.

Atrial fibrillation (AF) is a cardiac arrhythmia associated with various risk factors, including inflammation, and characterized by ectopic activity and reentrant substrates in the atria (Nso et al., 2021). The mechanism by which exosome blockade with GW4869 inhibits ferroptosis involves the modulation of microRNA content within exosomes, specifically miR-23a-3p (Figure 6). In AF pathogenesis, GW4869 inhibits exosome release, disrupting intercellular communication. This blockade reduces the delivery of miR-23a-3p, an important regulator of ferroptosis, from AF-affected cardiac fibroblasts (CFs) to cardiomyocytes. miR-23a-3p targets SLC7A11, a key component of the system Xc^−^ antiporter, suppressing its expression and leading to decreased levels of antioxidant proteins GPX4 and FTH1. This downregulation promotes ferroptosis and GW4869, by impeding exosome release, mitigates the inhibitory effect of miR-23a-3p on SLC7A11, preserving the cellular defense against ferroptosis in AF pathophysiology (Liu et al., 2022).

### 5.11 Inflammatory bowel disease (IBD)

Inflammatory bowel disease (IBD), comprising conditions like ulcerative colitis and Crohn’s disease, involves complex interactions between genetics, environment, and immune responses (Hu et al., 2022). Ferroptosis, an iron-dependent cell death process, plays a significant role in IBD pathogenesis, with excess iron triggering oxidative stress, lipid peroxidation, and cell death, contributing to intestinal epithelial damage. Inhibiting ferroptosis shows promise in ameliorating IBD symptoms, and key regulators like GPX4 and pathways such as NRF2/HO-1 and ER stress are implicated in this process (Xu et al., 2021). Human umbilical cord mesenchymal stem cell-derived exosomes (hucMSC-Ex) were characterized and investigated for their therapeutic potential in IBD using a mouse model. Treatment with hucMSC-Ex improved weight, reduced disease activity, and modulated inflammatory factors, indicating a protective effect on IBD. Mechanistically, hucMSC-Ex inhibited ferroptosis in IBD colon tissues, and this effect was mediated by miR-129-5p targeting ACSL4 (Figure 6). Transfection of hucMSC-Ex with miR-129-5p mimics further alleviated IBD symptoms, downregulated ferroptosis markers, and reduced inflammation (Wei et al., 2023).

### 5.12 Multiple sclerosis

Multiple sclerosis (MS), a prevalent neurological disorder affecting millions globally, leads to substantial declines in quality of life, physical function, and cognitive ability. Recently the role of ferroptosis in the pathogenesis of multiple sclerosis has been discussed, and the importance of ferroptosis inhibitors has been highlighted (Van San et al., 2023). Ferroptosis contributes to the demyelination process in MS by triggering oxidative stress and lipid peroxidation. Oligodendrocytes (OLs), which are particularly vulnerable to free radical attacks in MS, are the primary targets affected by this process. Iron deposits at inflammatory sites in the CNS, observed in MS and its animal model, are associated with oxidative damage to tissues, leading to demyelination and neurodegeneration (Li et al., 2022c). MSC-derived exosomes, containing miR-367-3p, protect against demyelination in MS by influencing ferroptosis. Upon internalization by susceptible microglial cells like BV2 microglia, these exosomes elevate intracellular miR-367-3p levels, countering its downregulation during erastin-induced ferroptosis. Specifically targeting the pivotal regulator EZH2, linked to neuroinflammation and demyelination, miR-367-3p alleviates its suppressive impact on SLC7A11, a critical component of the cysteine/glutamine antiporter system Xc^−^. This restoration of SLC7A11 function is crucial for maintaining cellular redox balance, orchestrating a protective mechanism against microglial ferroptosis and mitigating demyelination in the context of MS (Fan et al., 2023a).

### 5.13 Disc degeneration

Intervertebral disc degeneration (IDD) is a major contributor to lower back pain, with current treatments offering limited relief. Pathological changes in IDD involve extracellular matrix (ECM) degradation, apoptosis, senescence, and inflammation. A recent focus has been on ferroptosis, triggered by iron and ROS. Ferroptosis is linked to various degenerative disorders, and its role in IDD is emerging (Fan et al., 2023b). In the context of IDD, the ncRNA component, particularly circular RNAs present in extracellular vesicles derived from mesenchymal stem cells (MSC-EVs), assumes a critical role in mitigating ferroptosis. Specifically, circ_0072464, contained in these exosomes, functions as a ceRNA by competitively binding to miR-431. Through this molecular interaction, circ_0072464 effectively sequesters and neutralizes miR-431’s inhibitory influence on its downstream target, NRF2 (Figure 6). The heightened expression of NRF2, consequently, contributes to alleviating ferroptosis in nucleus pulposus cells (NPCs). This orchestrated interplay further influences the downstream expression of critical ferroptosis-related markers, with an observed increase in the expression of the antioxidant enzyme GPX4 and a concurrent decrease in the expression of the pro-ferroptotic protein ACSL4. The complex network of regulation involving ncRNAs within MSC-EV-derived exosomes provides a promising therapeutic approach for mitigating ferroptosis associated with IDD and fostering disc homeostasis (Yu et al., 2022).

### 5.14 Sepsis-induced lung injury

Lung injury resulting from sepsis represents a serious manifestation of multiple organ dysfunction syndrome, frequently culminating in the onset of acute respiratory distress syndrome (ARDS) (Jiao et al., 2021). ADSC exosomes alleviate ferroptosis in PMVECs exposed to LPS-activated macrophage conditioned medium, countering increased oxidative stress. Successfully isolated and delivered to PMVECs, these exosomes enhance viability, reduce apoptosis, and restore migration capability impaired by LPS-induced macrophage CM. Additionally, ADSC exosomes effectively attenuate ROS accumulation and mitigate oxidative injury in PMVECs. The protective effects involve upregulating the antioxidant enzyme GPX4 through the Keap1/Nrf2 signaling pathway following delivering miR-125b-5p (Jiao et al., 2021; Shen et al., 2023).

## 6 Conclusion

In conclusion, our exploration into the landscape of ncRNAs, with a particular emphasis on miRNAs, lncRNAs, and circRNAs, has unveiled a fascinating tapestry of regulatory mechanisms governing ferroptosis. The spotlight on exosomal ncRNAs, acting as messengers between cells, has illuminated their pivotal role in orchestrating ferroptosis across diverse pathological scenarios. NcRNAs, whether acting as promoters or inhibitors, modulate ferroptotic pathways, intersecting with key players like System Xc^−^ and GPX4. Turning our gaze to ischemic reperfusion injuries, the regulatory effects of exosomal miRNAs on ferroptosis in cardiac, renal, and cerebral tissues have been a focal point. Noteworthy miRNAs, such as miR-26b-5p and miR-140-5p, have been identified as critical players in influencing key regulators of ferroptosis, thereby shaping the outcome of myocardial and renal cells post-injury. These findings not only deepen our understanding of the molecular intricacies at play but also present avenues for interventions to mitigate the impact of I/R injuries. The exploration extends to liver fibrosis, where exosomal ncRNAs, particularly lncRNA WAC-AS1, wield influence over HSCs, modulating BACH2 and instigating ferroptosis. This dual role of ferroptosis in the fibrotic process adds a layer of complexity to our comprehension of liver pathology, with potential therapeutic implications. In essence, the diverse roles played by exosomal ncRNAs in modulating ferroptosis across various pathological conditions underscore their significance as potential therapeutic targets. The interconnectedness of these molecular players reveals a complex but finely tuned regulatory network that, when deciphered, holds promise for innovative therapeutic strategies. As we navigate the evolving landscape of molecular biology, the intersection of ncRNAs and ferroptosis presents an exciting frontier for future research and therapeutic exploration. Exosomal ncRNAs derived from MSCs sourced from various tissues, including bone, umbilical cord, adipose, peripheral blood, and urine, hold therapeutic potential for diverse human diseases. These exosomal ncRNAs exhibit the capability to modulate ferroptosis, either by suppressing or inducing it, offering promising avenues for disease intervention. Recent research suggests their efficacy in conditions marked by aberrant ferroptotic processes, such as neuronal injuries (e.g., MS and spinal cord injury), cardiovascular diseases (e.g., myocardial infarction and atrial fibrillation), kidney diseases (e.g., HBV-GN and renal ischemia), autoimmune diseases (e.g., IBD and MS), liver diseases (e.g., hepatic ischemia and liver fibrosis), and wound injuries (e.g., DFU and UVB-induced skin injury). MSC-derived exosomal ncRNAs act as critical mediators, influencing cellular responses and signaling pathways associated with ferroptosis regulation. The interplay between these exosomal ncRNAs and ferroptosis provides a foundation for targeted therapeutic strategies, opening up novel possibilities for precision medicine. Harnessing the regenerative and regulatory potential of MSC-derived exosomal ncRNAs from diverse sources represents a cutting-edge approach with broad implications for advancing personalized treatments and improving outcomes in a spectrum of human diseases.
